# Polar or nonpolar? That is not the question for perovskite solar cells

**DOI:** 10.1093/nsr/nwab094

**Published:** 2021-05-31

**Authors:** Boyuan Huang, Zhenghao Liu, Changwei Wu, Yuan Zhang, Jinjin Zhao, Xiao Wang, Jiangyu Li

**Affiliations:** Department of Materials Science and Engineering, Southern University of Science and Technology, Shenzhen 518055, China; Guangdong Key Laboratory of Functional Oxide Materials and Devices, Southern University of Science and Technology, Shenzhen 518055, China; Shenzhen Key Laboratory of Nanobiomechanics, Shenzhen Institute of Advanced Technology, Chinese Academy of Sciences, Shenzhen 518055, China; Shenzhen Key Laboratory of Nanobiomechanics, Shenzhen Institute of Advanced Technology, Chinese Academy of Sciences, Shenzhen 518055, China; Shenzhen Key Laboratory of Nanobiomechanics, Shenzhen Institute of Advanced Technology, Chinese Academy of Sciences, Shenzhen 518055, China; School of Materials Science and Engineering, Key Laboratory of Smart Materials and Structures Mechanics of Hebei Province, Shijiazhuang Tiedao University, Shijiazhuang 050043, China; Shenzhen Key Laboratory of Nanobiomechanics, Shenzhen Institute of Advanced Technology, Chinese Academy of Sciences, Shenzhen 518055, China; Department of Materials Science and Engineering, Southern University of Science and Technology, Shenzhen 518055, China; Guangdong Key Laboratory of Functional Oxide Materials and Devices, Southern University of Science and Technology, Shenzhen 518055, China; Shenzhen Key Laboratory of Nanobiomechanics, Shenzhen Institute of Advanced Technology, Chinese Academy of Sciences, Shenzhen 518055, China

**Keywords:** perovskite solar cells, polarity, ferroelectricity, first-principles calculations, scanning probe, photovoltaic implication

## Abstract

Perovskite solar cells (PSC) are promising next generation photovoltaic technologies, and there is considerable interest in the role of possible polarization of organic-inorganic halide perovskites (OIHPs) in photovoltaic conversion. The polarity of OIHPs is still hotly debated, however. In this review, we examine recent literature on the polarity of OIHPs from both theoretical and experimental points of view, and argue that they can be both polar and nonpolar, depending on composition, processing and environment. Implications of OIHP polarity to photovoltaic conversion are also discussed, and new insights gained through research efforts. In the future, integration of a local scanning probe with global macroscopic measurements *in situ* will provide invaluable microscopic insight into the intriguing macroscopic phenomena, while synchrotron diffractions and scanning transmission electron microscopy on more stable samples may ultimately settle the debate.

## Teaser

The debates regarding polarity of organic-inorganic halide perovskites and its photovoltaic implications were discussed from both the experimental and theoretical aspects, bringing us new insights into this question.

## INTRODUCTION

**Figure 1.**
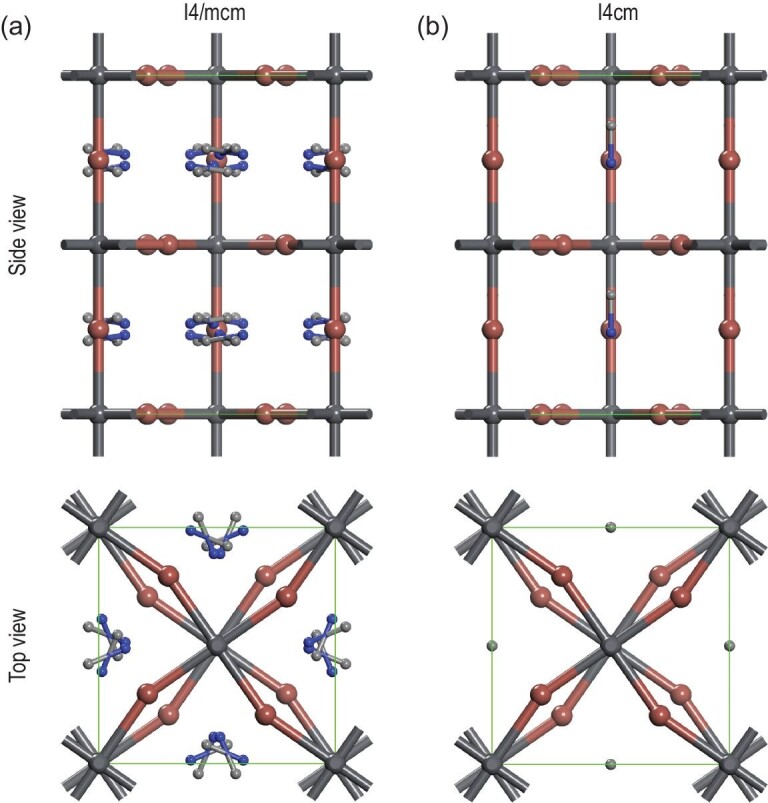
Schematic lattice of nonpolar I4/mcm (a) and polar I4cm (b) space groups for MAPI from side and top views. The hydrogen atoms are hidden for simplicity.

Ever since the spectacular rise of perovskite solar cells (PSCs), there have been suggestions on possible roles of ferroelectric polarization in their photovoltaic conversion. Perovskite materials, particularly oxides, are often ferroelectric, and early theoretical calculations indicated that polarization in organic-inorganic halide perovskites (OIHPs) may help charge separation and facilitate carrier transport [1]. However, the ferroelectricity of OIHPs has not been firmly established experimentally, and the possible polarity of OIHPs is still hotly debated [2,3]. There is considerable theoretical and experimental evidence to support either point of view [4]. Both nonpolar I4/mcm (Fig. 1a) and polar I4cm (Fig. 1b) space groups are possible for CH_3_NH_3_PbI_3_ (MAPI) [5–7], and the structural difference is very subtle, making it difficult to differentiate these by conventional structural characterization techniques such as diffraction. Indeed, the structure details of MAPI have not been fully resolved, and the poor stability of the materials exacerbates the problem. In this review, we examine recent literature on the polarity of OIHPs, and argue that they can be both polar and nonpolar, depending on composition, processing and environment. Implications to photovoltaic conversion, especially hysteresis, are also discussed.

## THEORETICAL CONSIDERATIONS

Differing from traditional perovskite, the component at the A site in OIHP is positioned by a molecule-type ion, which may have an intrinsic dipole and induce the deformation of the octahedron framework caused by the interatomic hydrogen bond. Therefore, the apparent polarization of OIHP is the collective polarization of each unit impacted by the orientation of the A-site molecule. In the case of MAPI, the major structural difference between the polar I4cm and nonpolar I4/mcm phases is the orientation of MA cations, which have an intrinsic dipole of ∼2.3 D [1]. In the polar phase, the C−N dipole shows a ‘head-tail’ alignment along the c axis and displays a large polarization of several *μ*C/cm^2^ [8–11], whereas in the nonpolar counterpart, because of space group symmetry, each MA cation is usually described with partial occupancies with four identical positions and thus exhibits no net polarization [12]. Nevertheless, the orientation of an MA cation can distort the neighboring iodides from their centrosymmetric positions, leading to ferroelectricity [13].

The optimal orientation of MA in MAPbI_3_ bulk has not yet been determined through theoretical models and methods. Brivio *et al.* calculated the total energy of MA arrays along <100>, <110> and <111> directions in the cubic phase and found that <100> is the most stable orientation with energy difference less than 15 meV per atom [14]. Bechtel *et al.* calculated the full energy landscape for rigid-body rotations and translations of MA in the cubic phase and reached the same conclusion. They revealed that the preferential orientation is attributed to the strong N-H…I interactions between MA and the Pb-I framework along the <100> direction [15]. However, others reached different conclusions. Shimamura *et al.* used a cubic symmetry-assisted analysis and found that the prominent orientation of MA is in crystalline <110> directions, rather than the <100> and <111> directions [16]. Xu *et al.* studied MA orientation using the swarm intelligence-based structure prediction method combined with DFT calculations, but they found that the <012> orientation was most stable rather than the aforementioned directions [17].

Despite the puzzling optimal orientation, there is agreement that the orientation of MA tunes the strength and direction of the hydrogen bond between MA^+^ and I^−^, which is rather weak (∼0.09 eV/cation) [18]; there is only slight energy difference (< 0.1 eV/unit) between the two phases and the phase transition barrier is also quite small (about 0.2 eV/unit) [9]. Such a tiny difference makes for an easy transition between the polar and nonpolar phases at room temperature [19,20]. Furthermore, the subtlety between the two phases also makes the debate regarding polar and nonpolar nature of OIHPs notably dependent on method, model, size and time-scale in ab initio calculations [9,21]. We should not only focus on the origin of the polarity in its primitive cell, but also the long-range dynamics of the MA cations in a wider vision. The ab initio molecular simulation is a versatile method that can consider more operational conditions (such as temperature, long-range dynamics, etc.) with accuracy. The random order of MA and the phase transition between the two phases have been tracked, usually indicating an antiferroelectric nature of tetragonal OIHPs [19,20,22].

**Figure 2.**
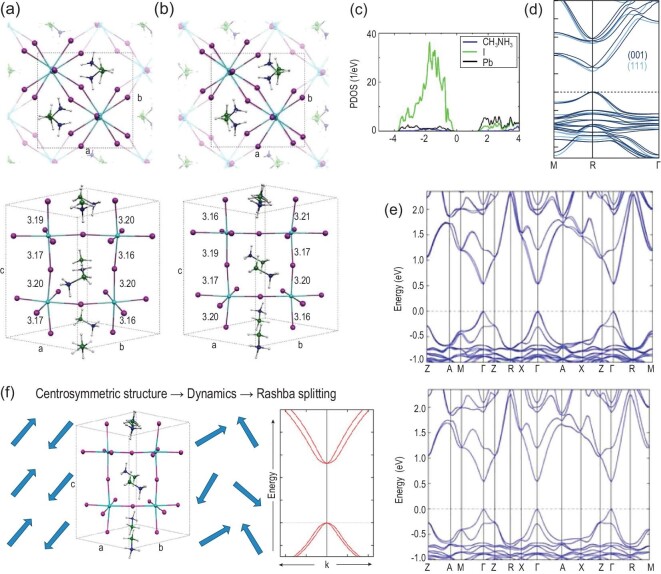
Theoretical studies on photovoltaic properties of polar and nonpolar MAPI in bulk phase. The top and side views of (a) polar and (b) nonpolar phases of MAPI relying on the orientation of MA cation. The Pb, I, C, N, H atoms are colored in light blue, pink, blue, green and white. (c) Projected density of states of MAPI. The Pb and I instead of MA group mainly contribute to the CBM and VBM. (d) Band structure of MAPbBr_3_ (MAPB) with MA along different directions. A direct to indirect band transition is present when the orientation of MA changes from <111> to <001> direction. (e) The Rashba/Dresselhaus effect in the polar phases. Band splitting is present near the CBM and VBM in the polar phase, while in the nonpolar phase, the Rashba/Dresselhaus effect does not exist. (f) The dynamic Rashba effect in MAPI caused by random rotation of MA. Adapted with permission from Refs [9,24,28,31].

As mentioned, the phase transition causes reorientation of the MA cation and changes the hydrogen bond, which is very weak and has little contribution to the valence band maximum (VBM), conduction band minimum (CBM) or even band gap (∼0.1 eV fluctuation) [23,24] (Fig. 2c). However, the influence of the collective behavior of MA dynamics on the band structure cannot be neglected, as this will influence the photoelectric performance. Geng *et al.* designed several MA orientations in a supercell and tracked the band gap of MAPI. Their theoretical results showed that the band gap is tunable, ranging from 1.3 to 1.6 eV [25]. Mosconi *et al.* performed ab initio molecular dynamics simulations and also found a variation of ±0.1 to 0.2 eV of the electronic properties with the ion dynamics [22], which is consistent with Mladenović’s works [26]. Besides the value of the band gap, the orientation of MA can also cause transition from direct band gap to indirect band gap. Motta *et al.* performed van der Waals-corrected DFT calculations and revealed that the band gap will become indirect if MA orients along a <011>-like direction, causing dynamic change of the band structure which might be the origin of the slow carrier recombination of MAPI [27]. Later they found a similar direct–indirect transition in MAPbBr_3_ (MAPB). Their DFT calculations demonstrated that MAPB is a direct band gap semiconductor when MA is oriented along the <111> direction but turns indirect along the <100> direction (Fig. 2d) [28].

**Figure 3.**
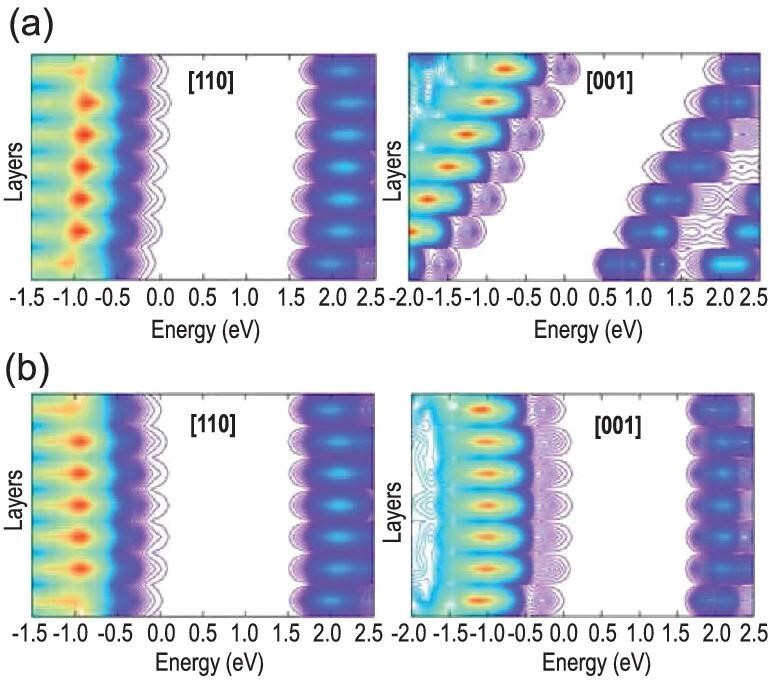
Density of states (DOS) of the valence and conduction bands for the surface constructed from (a) polar and (b) nonpolar phases along [110] and [001] directions. Adapted with permission from Ref. [9].

**Figure 4.**
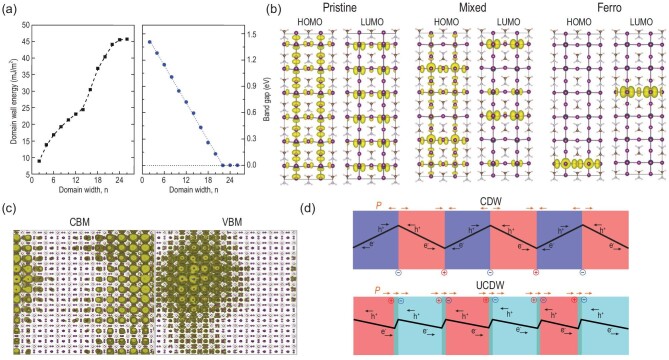
Theoretical studies on the properties of the domain wall in MAPI. (a) Domain wall energy and electronic band gap as a function of domain width in MAPI. (b) The highest occupied molecular orbital (HOMO) and lowest unoccupied molecular orbital (LUMO) charge densities in pristine MAPI, mixed and ferro systems. (c) The charge density of the CBM and VBM states in the MAPI supercell with MA randomly oriented. Both the CBM and VBM charge densities are strongly localized. (d) The charged (top) and uncharged (bottom) domain walls formed by the orientation of MA cation. Head-to-head and tail-to-tail charge domain walls attract electron and hole, respectively, facilitating charge separation. Adapted with permission from Refs [36–39].

The Rashba/Dresselhaus effect is a phenomenon in solid-state physics in which spin-orbit interaction causes energy bands to split, especially in a crystal system lacking inversion symmetry. The polar OIHP is a typical case in which to present such effect. In the I4cm polar phase (shown in Fig. 2a), the Rashba effect can be detected by ab initio calculations, resulting in splitting of frontier orbitals near Fermi level along the M-Γ-Z direction and creation of an indirect band gap (Fig. 2e) [9,29], while in the I4/mcm nonpolar phase (Fig. 2b), the Rashba effect does not exist (Fig. 2e) [12]. Niesner *et al.* used angle-resolved photoelectron spectroscopy and detected the Rashba/Dresselhaus effect in MAPB [30], which is consistent with theoretical prediction. Furthermore, a ‘dynamic Rashba effect’ was proposed by Etienne *et al.* through the rotation of MA or the deformation of the framework when the thermal movement of MA was tracked by van der Waals-corrected ab initio simulations (Fig. 2f) [31]. Such an effect might lead to reduced recombination rate caused by a spin-forbidden transition [32]. Niesner *et al.* resonantly excited photocurrents in single-crystalline tetragonal MAPI with circularly polarized light to clarify the existence of such effect. Further studies showed that the energy splitting between the spin-polarized transition and the direct optical transition, as well as the amplitude of the circular photogalvanic effect, increased with temperature [33]. Wu *et al.* used a broad range of temperature-dependent and time-resolved optical spectroscopies, correlated with density functional theory (DFT) and molecular dynamics (MD) calculations and electrical characterizations, and proved the existence of indirect tail states below the direct transition edge in MAPB arising from a dynamical Rashba splitting effect [34]. Recalling the general features of Rashba/Dresselhaus splitting, Kepenekian *et al.* used symmetry analysis and DFT calculations and discussed the possibility of designing spintronic devices. They found even in the centrosymmetric system, the Rashba effect can be present under the external electric field [35]. The polarization can also impact electronic properties of the surface structure apart from the bulk. The orientation of MA cations can give rise to strong bending in the valence and conduction bands of polar phases, as exhibited by a gradient in density of states (DOS) along the [001] direction (Fig. 3a). Such band bending may reduce the carrier recombination and assist charge separation [9]. For the nonpolar phase (Fig. 3b), on the other hand, DOS along both [110] and [001] directions are nearly constant. In the mesoscopic or macroscopic system, the domain wall can be formed in OIHP and display different electronic properties compared with the bulk. Chen *et al.* studied the formation and band gap vs the domain width. As shown in Fig. 4a, they reported that the domain is stable with rather low formation energy and that increasing the domain width can decrease the electronic band gap from ∼1.4 eV to 0 [36]. The MA orientation can tune the charge aggregation near CBM and VBM [37] (shown in Fig. 4c), which might act as the ‘ferroelectric highway’ and profit the carrier separation [1]. The polarization in ferroelectric domains can suppress the nonradiative electron-hole recombination based on the time-domain ab initio study (Fig. 4b) [38]. Here, the pristine system is pure I4cm polar phase with aligned C−N bonds, the mixed system refers to a mixture of aligned and anti-aligned C−N bond pairs, presenting nonpolar characteristics, while the ferro-system contains two domains with opposite C−N polar bonds. Charge separation is clearly observed in mixed and ferro-systems with opposite polar axes, beneficial for recombination suppression. Furthermore, when the domain wall is charged, the band gap can be reduced by 20–40%, and there is a strong potential step that facilitates electron-hole separation (Fig. 4d), providing segregated channels for photoexcited charge carriers [39], which are desirable for high conversion efficiency [1]. Summarizing these theoretical studies, there is general agreement that polarization may be beneficial for photovoltaic conversion.

## EXPERIMENTAL EVIDENCE

**Figure 5.**
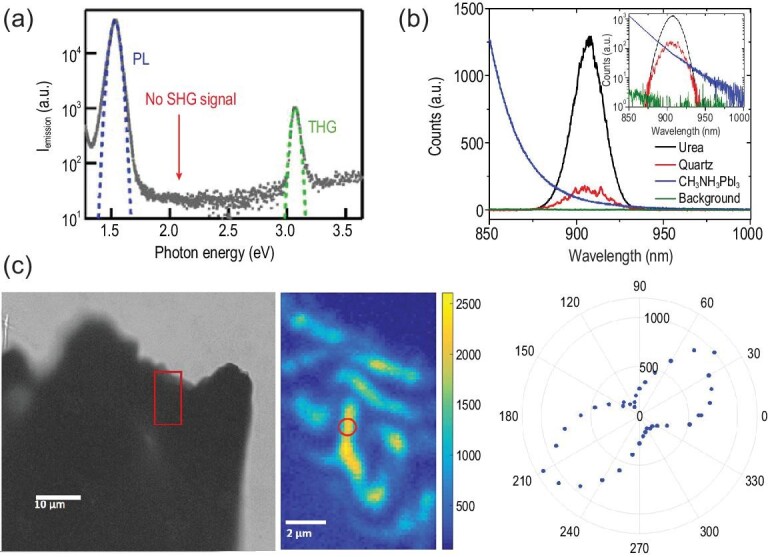
Second harmonic generation (SHG) response. (a) Emission spectra of MAPI under excitation at 1.03 eV. (b) Spectra of second harmonic generated at 900 nm, with incident 1800 nm laser pulse measured on urea (in black) at 1.75 mW incident power, quartz (in red) at 14.9 mW, and MAPI (in blue) at 14.9 mW after subtracting the detector background, which is shown in green. (c) A polar plot of the SHG signal (right) from the marked point in the middle SHG mapping, the area of which is approximately marked by the red box in the bright-field transmission image (left) of a crystal fragment. Adapted with permission from Refs [62,67,76].

Given the uncertainty associated with two possible tetragonal lattices for MAPI (Fig. 1), it was natural to carry out detailed structure analysis via X-ray and neutron diffraction techniques [40–45]. However, the subtle structural difference has proven difficult to resolve, and the data can be fit by either polar [20,38,46–58] or nonpolar [8,19,59–68] space groups. Attempts have also been made using transmission electron microscopy (TEM) [69–71], although the materials are prone to degradation [72] and it is virtually impossible to get an atomically resolved image with one exception, wherein an HRTEM image acquired from more stable MAPbBr_3_ (MAPB) showed polar domains [73]. As a result, much effort has been devoted to functional probing, as the properties of polar and nonpolar groups are drastically different. Macroscopic ferroelectric, pyroelectric and dielectric measurements have also been carried out [48,50,59,60,64,66,74–76], although leakage current and ionic migration often make the data interpretation ambiguous. While polar structure, with the breaking of inversion symmetry, is expected to be active in optical second harmonic generation (SHG) [12], the experimental data are inconclusive because of the strong background from other nonlinearities [62,77]. Absence of macroscopic SHG was first reported by Yamada *et al.* (Fig. 5a) [67,77], who did not observe any SHG signal under excitation at 1.03 eV (1204 nm) after application of a poling electric field around 1 kV/cm to the sample, while third harmonic generation and PL signals were clear because of two-photon absorption. To exclude the possibility that the second harmonic generated at wavelengths <800 nm would be strongly absorbed by MAPI in view of its small band gap, Govinda *et al.* adopted 1800 nm to perform SHG experiments, but the absence of a SHG response at 900 nm was still evident (Fig. 5b) [62]. It remains possible that ferroelectric domain size is below laser wavelength. Indeed, spatially resolved SHG mapping (Fig. 5c) provided strong evidence on polar domains in MAPI [76], and local polarity can be averaged out at macroscopic scale, which highlights the importance of spatially resolved local probing. Piezoresponse force microscopy (PFM) is a powerful tool to probe local electromechanical coupling at the nanoscale [78,79], and it has been widely applied to study OIHPs. Not surprisingly, PFM data reported largely fall into two categories, supporting either polar [46,47,49–54,56–58] or nonpolar [8,61,63,65,68,80] structure. In fact, even with quite similar experimental observations, for example characteristic lamellar domain patterns reported by different groups [53,81], the interpretations can be completely opposite. This is because electromechanical responses as probed by PFM can arise from complex microscopic mechanisms [82], especially ionic activities, making PFM data analysis for OIHPs nontrivial. This is best illustrated by the recent debates in *Nature Materials* [2,3] on the chemical nature of ferroelastic domains in MAPI reported by Liu *et al.* [68], and there is no agreement on whether it is ferroelectric or not. The latest publication from Liu *et al.*, however, raised an alternative interpretation, that chemical and strain gradients induce flexoelectric polarization in MAPI [83]. This latest study seems to suggest symmetry breaking in MAPI more aligned with polar structure, although its microscopic origin is different.

**Figure 6.**
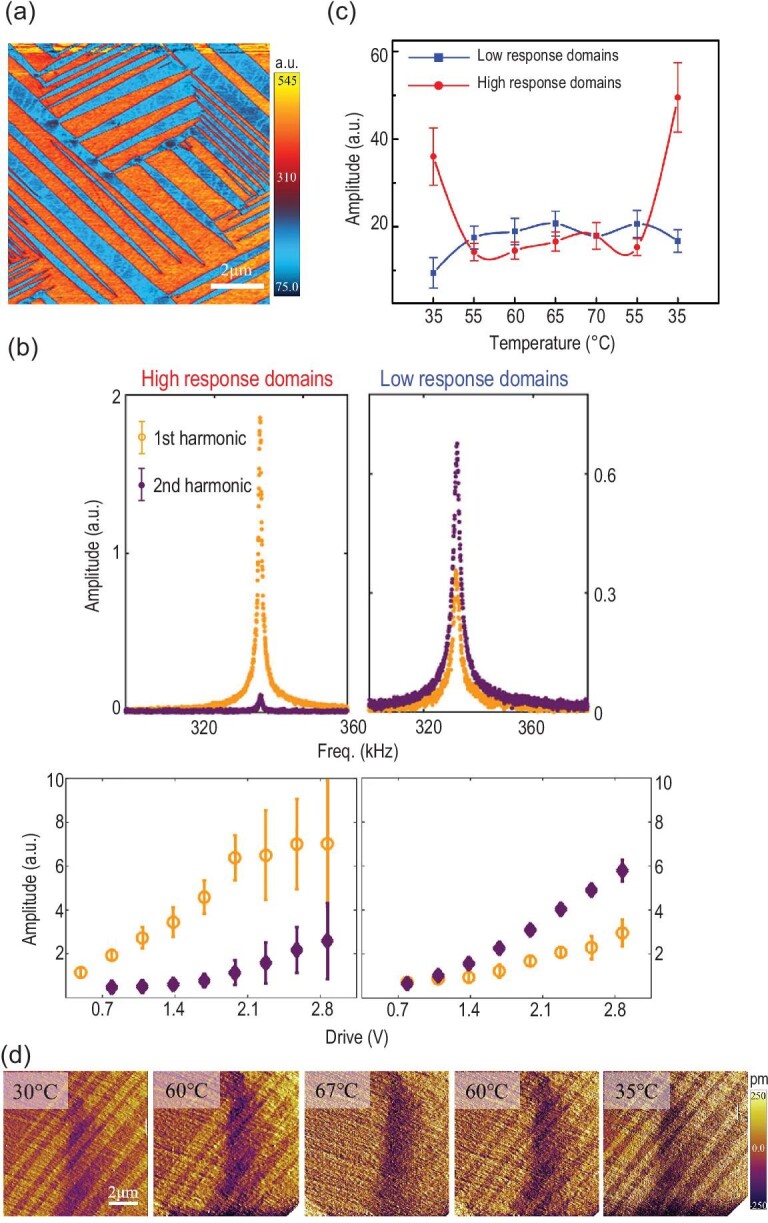
Alternating polar and nonpolar domains in MAPI. (a) Ferroic domain patterns of a MAPI crystal revealed by the vertical PFM. (b) The first row: point-wise tuning of piezoresponse versus frequency showing a point in high-response polar domain has dominant first harmonic response and negligible second harmonic one, while a point in low-response nonpolar domain has higher second harmonic response; the second row: comparison of first and second harmonic responses versus alternating current voltages averaged over a number of points in high- and low-response domains. (c) Piezoresponses averaged in high-response polar and low-response nonpolar domains showing opposite trend with respect to temperature, yet convergence beyond phase transition. (d) AFM topography mappings under a sequence of temperature across phase transition showing appearance and reemergence of ferroic domains. Adapted with permission from Ref. [4].

In 2018, we reported an in-depth PFM study [4] on single crystalline MAPI [84], with the goal to resolve the microscopic mechanisms of piezoresponse probed. We acquired the most compelling domain patterns (Fig. 6a), and established distinct mechanisms underlying the piezoresponse in adjacent domains (Fig. 6b) suggesting the coexistence of alternating polar and nonpolar structures. In particular, polar domains exhibit predominantly first harmonic linear response that arises from piezoelectricity, while nonpolar domains possess predominantly second harmonic quadratic response arising from ionic motion induced electrochemical dipoles [85]. This interpretation is supported by the drastically different thermal variation of piezoresponses in polar and nonpolar domains, one increasing with temperature, the other decreasing with temperature (Fig. 6c), which converge above cubic-tetragonal transition temperature. When the temperature is reduced, the original domain configuration is recovered (Fig. 6d), demonstrating a strong memory effect. In our view, this set of data unambiguously establishes alternating polar and nonpolar domains in our crystal, and this observation can reconcile all the inconsistent experimental data and theoretical analysis reported in the literature. Other PFM studies rarely examine the linear versus quadratic piezoresponses, and thus it is difficult to identify the dominant mechanisms critical for the differentiation. Theoretical calculation suggested that the energetic difference between polar and nonpolar lattice is tiny, <100 meV [9], and thus depending on composition, processing and environment, the balance can be easily tipped, making both structures possible in experiments. As summarized in Table 1, OIHPs with various compositions are reported to be either polar or nonpolar [5,6,12,19,39,41,42,44,45,47–60,62–68,73,76,83,86–101]. In addition, the processing methods may also affect the polarity of MAPI. A comparison of the representative preparation methods for MAPI reported to be polar [52–54,57] and nonpolar [60,68,102] presents that treating MAPI with dimethylformamide (DMF) vapor on a hotplate after general synthesis procedure and inclusion of methylammonium chloride (MACl) or PbCl_2_, in which chlorine was shown to be beneficial for obtaining MAPI with large grains [103], together with methylammonium iodide (MAI) during synthesis might be favorable for forming polar MAPI. In a sense, we all are both wrong and right that OIHPs can be both polar and nonpolar.

**Table 1.** Literature survey on the polarity of OIHPs with various compositions.

Composition	Non-polar	Polar

MAPbCl_3_	Refs [66,86]	Ref. [39]
MAPbBr_3_	Refs [19,66,87–89]	Refs [39,73,90]
MAPbI_3_	Refs [5,12,19,42,44,45,59,60,62,68,91]	Refs [6,41,47–58,76,83,92]
FAPbBr_3_		Ref. [94]
FAPbI_3_	Ref. [95]	Ref. [93]
CsPbCl_3_	Refs [96–98]	
CsPbBr_3_	Ref. [99]	Refs [100,101]
CsFAMAPbI_x_Br_3−x_	Ref. [63]	

If MAPI is polar, can its polarity be switched? Macroscopically this is difficult to resolve, as the data are often smeared by leakage current, ionic migration as well as spatial averaging. Nevertheless, a number of recent reports indicate that electric field can indeed manipulate the domain structures [47,54,104], pointing toward a ferroelectric nature of the domain. The unambiguous switching of MAPI domains, however, requires further studies. We also note that ferroelectricity has been reported in MAPB [90], CsPbBr_3_ [101] and mixed perovskites [93,105,106].

## PHOTOVOLTAIC IMPLICATIONS

**Figure 7.**
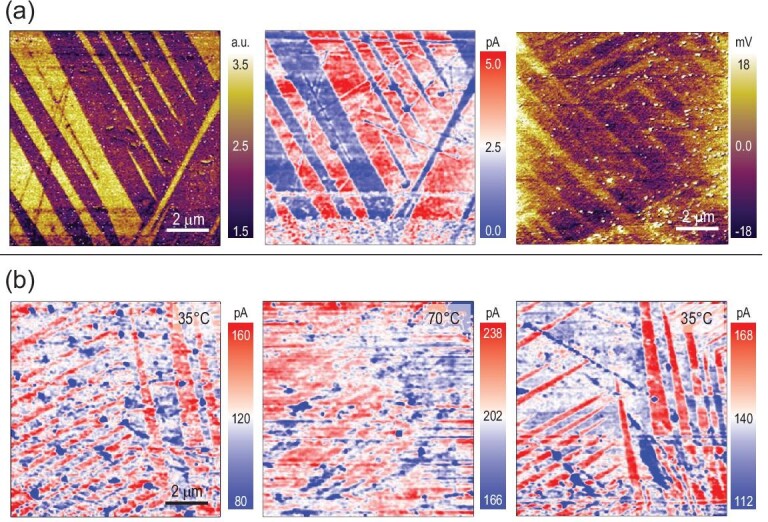
Regulation of photocurrent by polar domains. (a) Correlation between PFM (left) and photocurrent (middle) of the same area with reduced photocurrent in polar domains; and surface potential distribution under light follows ferroic domain pattern in Fig. 6a with negatively shifted potential in polar domains. (b) Photocurrent distribution in a separate domain pattern at different temperatures across phase transition, showing the disappearing domain pattern at 70°C upon heating and its reemergence at 35°C after cooling. Adapted with permission from Ref. [4].

It is also important to examine the implications of OIHPs’ polarity, or lack of it, to photovoltaic conversion, otherwise the problem remains largely academic. We have indeed observed photo-induced domain switching in MAPI via PFM [58], and a similar phenomenon has been observed under photo-excited scanning tunneling microscopy (STM) [107]. The light-domain interactions have been studied by Liu *et al.* [91,108], and poling has been shown to shift diode characteristic of MAPI [54]. Furthermore, piezoelectric modulations of photocurrent have also been observed [109,110]. All these studies suggest that polar structure may influence the photovoltaic conversion process, and to the very least, band bending induced by spontaneous polarization can either promote or hinder carrier transport, depending on its direction. Our study in 2018 indeed revealed that a polar domain possesses smaller photocurrent compared to a nonpolar one [4] (Fig. 7a), and upon heating and cooling across phase transition, a memory effect in photocurrent analogue to ferroic domains is also observed (Fig. 7b), confirming modulation of photocurrent by domains.

Nevertheless, there remains controversy about the effect of polarization on photovoltaic hysteresis. Unfavorable hysteresis is usually observed in the current-voltage (*I*−*V*) curve at various scanning rates or directions [111], casting doubts on the validity of the performance of solar cells and making it hard to compare stability data among them. Despite booming research and significant progress on the efficiency of perovskite solar cells, fundamental understanding of frequently observed hysteresis is still inadequate.

Among various interpretations of hysteresis, ferroelectricity was suggested as one plausible origin at the very beginning. For example, Wei *et al.* attributed the hysteresis to the ferroelectric effect and built a ferroelectric diode model to explain the dependence of hysteresis on the scan range as well as the velocity [112]. They pointed out that special attention should be paid to optimization of power conversion efficiency. Recently, Ma *et al.* investigated correlations between the interfacial ferroelectricity and the hysteresis of specific heterojunctions by simulations [113]. They found that ferroelectricity is suppressed at the FAPbI_3_/TiO_2_ and MAPbI_3_/phenyl-C61-butyric-acid-methyl-ester (PCBM) interfaces. The substitution of strong polar MA (dipole moment: 2.29 D) by weak polar FA ions (dipole moment: 0.29 D) and interface passivation could eliminate the interfacial electric field between perovskite and TiO_2_, leading to consistent interfacial electronic dynamics and the absence of hysteresis [113]. Although it is now generally accepted that ions play a more important role in hysteresis [60,114,115], the separation of ionic effect and polar order is not trivial. For example, it has been reported that a dipolar Frenkel pair can be induced by ionic migration [116]. Meloni *et al.* claimed that hysteresis results from polarization of halide ion (vacancy) migration in the perovskite layer under the influence of the built-in and applied potential. The mobility of the other possible ionic species (MA^+^ and Pb^2+^) is much lower and not expected to give any significant contribution to polarization of devices [114]. We also found that while illumination may enhance polar response in Cs_0.05_FA_0.81_MA_0.14_PbI_2.55_Br_0.45_ (CsFAMA), only small photovoltaic hysteresis is observed at both the nano- and macroscale, demonstrating that the presence of strong polarization plays a negligible role in photovoltaic hysteresis. Based on multi-harmonic measurements, our study supports the concept that the primary mechanism responsible for photovoltaic hysteresis is ionic migration rather than polarization for this material [117].

## CONCLUSION AND OUTLOOK

Theoretical calculation is a versatile tool to reveal the interaction of MA with the Pb-I framework, and study the influence of MA orientation on optoelectronic properties at the nanoscale. Adequate achievements have been reported and some common views have been reached: (i) the orientation of MA is determined by the hydrogen bond and usually faces towards the low-index direction; (ii) the orientation of MA can cause deformation of the Pb-I framework because of the H…I hydrogen bond, which can break the symmetry of the system and cause polarization; (iii) just tuning the orientation of MA, namely, polarization or not has little influence on the value of band gap, can cause direct-indirect band transition, as well as the Rashba/Dresselhaus effect or even dynamic Rashba/Dresselhaus effect, which may reduce the recombination of carrier, increase the carrier lifetime and enhance the optoelectronic performance.

Experimental observations on the ferroic properties of perovskite solar cells were systematically reviewed, along with photovoltaic implications: (i) a subtle difference between polar and nonpolar structure cannot be resolved by diffraction techniques, TEM, conventional macroscopic measurements and SHG in a conclusive way because of sample damage or an averaging effect; (ii) a powerful scanning probe can capture spatially resolved functional response from different structures, although caution must be exercised to distinguish complex microscopic mechanisms among polarity, ionic motion and defect; (iii) modulation of photocurrent by polar and nonpolar domains is confirmed, while ions may play a more important role in hysteresis, which is crucial for the performance of solar cells.

**Figure 8.**
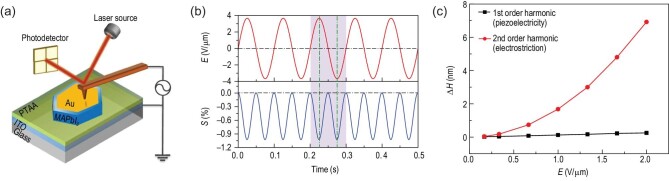
Electrostrictive response of MAPI single crystal. (a) Schematic illustration of AFM measurement of strain induced by electric field. (b) Electrostrictive strain of a 40 μm MAPI single crystal under a.c. bias at 10 Hz. (c) Thickness change measured by Mach-Zehnder interferometer resulting from first-order piezoelectricity and second-order electrostriction for a 40 μm MAPI single crystal under 100 Hz a.c. with different field amplitudes. Adapted with permission from Ref. [116].

We may find that polarization, whatever its exact origins, plays only marginal roles in PSCs, but the endeavor often brings in unexpected twists. As shown in Fig. 8, giant electrostriction has been reported [116], which was attributed to defect dipoles of Frenkel pairs induced by ionic migration. Here, it seems impossible to distinctly separate ionic migration, defect and polarity, all of which will be reflected in the experimental observations.

Although the beam-induced damage of synchrotron diffraction and scanning transmission electron microscopy on perovskite samples is inevitable, with continuous improvement in characterization as well as material stability [118–121], it is hoped that these techniques will ultimately settle the debate as shown in Table 2, by resolving the structure details of OIHPs. For example, Breternitz *et al.* recently presented crystallographic evidence that the symmetry breaking on MAPI comes from interaction of polar cation MA with the anion framework via synchrotron diffraction [13]. Besides, tentative efforts have been made to mitigate the damage for acquiring atomically resolved imaging [118], including but not limited to using cryo-conditions for higher dose tolerance of sample [122], increasing acceleration voltage to decrease radiolysis [123], and taking advantage of facet-dependent electron beam sensitivity [119]. Although further investigation is required to examine their validity, these methods have provided promising directions for future characterizations. In addition, macroscopic techniques, such as impedance spectroscopy [124,125], in combination with modeling and simulation, may provide valuable supporting data on the microscopic mechanisms. In this regard, integrating a local scanning probe with global macroscopic measurements *in situ* will provide invaluable microscopic insight into the macroscopic phenomena, which we are trying to develop, and it is particularly important to examine different and often competing dynamical processes from local relaxation studies [126]. From a theoretical perspective, as the energy difference between the polar and nonpolar phases is tiny and might varies with different functionals or methods, calculations with higher accuracy should be performed. In parallel, a study on the polarization should proceed not only microscopically but also mesoscopically considering the long-range interaction of the ferroelectric domains. Therefore, ab initio calculations of the larger-scale system are also needed. To mimic the real experimental conditions, other factors including temperature, strain and light should also be taken into account to investigate the dynamics of OIHP. Combined with the machine learning and artificial intelligence algorithm [127], the classical molecular dynamic simulation with accurate potential energy surface also needs to be improved. So are OIHPs polar or nonpolar? That might not be the question, but efforts made to answer it continue to deliver new insights.

**Table 2.** Literature survey on the polarity of OIHPs.

Technique	Nonpolar I4/mcm	Polar I4cm	Noncommittal

X-ray and neutron diffractions	Refs [5,42–45]	Refs [6,41]	Ref. [40]
Optic SHG	Refs [12,19,62,67]	Refs [76,93]	
Macroscopic measurements	Refs [59,60,64]	Refs [48,50,76,90,101]	Ref. [75]
Microscopic PFM	Refs [8,63,65,68,91]	Refs [46,47,49–58,83]	Refs [61,80,81,104]
TEM		Ref. [73]	Ref. [70]
DFT and MD simulations	Refs [12,19,66]	Ref. [92]	Refs [8,21]
